# SATISFACTION WITH WALK ON! FROM PROGRAM CLIENTS AND LEADERS

**DOI:** 10.1093/geroni/igad104.1694

**Published:** 2023-12-21

**Authors:** Rahma Ajja, Elizabeth Chmelo Kemp, Jaime Hughes, Lauren Van Laethem, Kimberly Welsh, Tami Guerrier, Mark Hirsch, Barb Nicklas

**Affiliations:** Wake Forest University School of Medicine – Winston-Salem, North Carolina, Winston Salem, North Carolina, United States; WFSM, Winston Salem, North Carolina, United States; Wake Forest School of Medicine, Winston-Salem, North Carolina, United States; Atrium Health Carolinas Rehabilitation, Charlotte, North Carolina, United States; Atrium Health Carolinas rehabilitation, Charlotte, North Carolina, United States; Atrium Health Carolinas Rehabilitation, Charlotte, North Carolina, United States; Atrium Health Carolinas Rehabilitation, Charlotte, North Carolina, United States; Wake Forest School of Medicine, Winston Salem, North Carolina, United States

## Abstract

This presentation demonstrates the high degree of satisfaction with Walk On! from qualitative survey data from both clients and program leaders. As part of the curriculum, each attendee completes a survey asking about satisfaction around location, day/time, length of program, and changes in walking abilities. The survey also includes an opportunity for open-ended responses and suggestions for improvement. Overall, surveys showed a very high degree of satisfaction with the program with 80-100% of clients indicating they are “Very Much” satisfied with the program components. All open-ended responses were positive and examples include: “Very pleased with the instruction and environment”; “Encouraged me to walk at home and neighborhood”; “This program gave me energy to walk better and has changed my life”; “Enjoyed the fellowship with others”; and “I now walk with more confidence”. Leaders also complete a satisfaction survey upon completion of their Leader Training program and after leading a Walk On! program. A majority of the leaders “Strongly Agreed” or “Agreed “that: The length of the training was appropriate (83%); Course materials were explained well (100%); Course materials were easy to use (100%); I feel prepared to lead Walk On! (67%). Leader statements include: “I loved everything about this program. It was not just a walking program to me. I had social, cognitive and physical growth” and “This program afforded me the opportunity to have a real collaborative, immersive experience with elders.” Together, these positive findings support the need to scale and spread WalkOn! into additional community settings.

